# Integrating clinicians, knowledge and data: expert-based cooperative analysis in healthcare decision support

**DOI:** 10.1186/1478-4505-8-28

**Published:** 2010-09-30

**Authors:** Karina Gibert, Carlos García-Alonso, Luis Salvador-Carulla

**Affiliations:** 1 Department of Statistics and Operations Research Universitat Politècnica de Catalunya, Barcelona, 08034, Spain; 2 Knowledge Engineering and Machine Learning Group, Universitat Politècnica de Catalunya, Barcelona, 08034, Spain; 3 ETEA Business Administration Faculty, University of Córdoba, Córdoba, 14004, Spain; 4 Department of Neurosciences. University of Cadiz. Plaza Falla 9 11003 Cadiz, Spain; 5 PSICOST Research Association. Plaza San Marcos 6. Jerez 11403, Spain

## Abstract

**Background:**

Decision support in health systems is a highly difficult task, due to the inherent complexity of the process and structures involved.

**Method:**

This paper introduces a new hybrid methodology *Expert-based Cooperative Analysis *(EbCA), which incorporates explicit prior expert knowledge in data analysis methods, and elicits implicit or tacit expert knowledge (IK) to improve decision support in healthcare systems. EbCA has been applied to two different case studies, showing its usability and versatility: 1) Bench-marking of small mental health areas based on technical efficiency estimated by *EbCA-Data Envelopment Analysis (EbCA-DEA)*, and 2) Case-mix of schizophrenia based on functional dependency using *Clustering Based on Rules (ClBR)*. In both cases comparisons towards classical procedures using qualitative explicit prior knowledge were made. Bayesian predictive validity measures were used for comparison with expert panels results. Overall agreement was tested by Intraclass Correlation Coefficient in case "1" and kappa in both cases.

**Results:**

EbCA is a new methodology composed by 6 steps:. 1) Data collection and data preparation; 2) acquisition of "Prior Expert Knowledge" (PEK) and design of the "Prior Knowledge Base" (PKB); 3) PKB-guided analysis; 4) support-interpretation tools to evaluate results and detect inconsistencies (here *Implicit Knowledg *-IK- might be elicited); 5) incorporation of elicited IK in PKB and repeat till a satisfactory solution; 6) post-processing results for decision support. EbCA has been useful for incorporating PEK in two different analysis methods (DEA and Clustering), applied respectively to assess technical efficiency of small mental health areas and for case-mix of schizophrenia based on functional dependency. Differences in results obtained with classical approaches were mainly related to the IK which could be elicited by using EbCA and had major implications for the decision making in both cases.

**Discussion:**

This paper presents EbCA and shows the convenience of completing classical data analysis with PEK as a mean to extract relevant knowledge in complex health domains. One of the major benefits of EbCA is iterative elicitation of IK.. Both explicit and tacit or implicit expert knowledge are critical to guide the scientific analysis of very complex decisional problems as those found in health system research.

## Background

Research methods in medicine are mainly based on a restrictive experimental approach. As an example, exclusion and inclusion criteria are defined in randomized control trials to reduce the complexity of the phenomena observed, while expert knowledge is disregarded as it is considered that using expert opinion introduces subjectivity into a scientific process. This approach has undoubtly contributed to the development of knowledge in medicine and it still constitutes the core of evidence-based healthcare [1], but systematically disregards also the well established parts of the corpus doctrinae which do not involve particular opinions, but just well consensued knowledge, which obviously exist. The overall value of the restrictive experimental approach may decrease as the levels of complexity of the analysed phenomenon increases; particularly in highly complex situations as those occurring when organisations behave as a complex adaptive system [2].

Healthcare systems involve very complex structures and high-dimensional interactions among multiple factors, where the *meaning *of the measurements becomes crucial for proper interpretation, and where phenomena are very difficult to model in their globality under classical approaches. Paradoxically, the complexity and non-linearity of health systems has not been acknowledged until very recently by international organisations such as WHO [3]. This omission has had major implications to data-analysis, too often based on techniques and procedures designed for linear phenomena, which should not be applied to systems characterised by non-linearity, self-organisation and constant change, fragmented but highly interconnected, history-dependent and counter-intuitive [3].

Although very accurate models can be obtained under classical approaches while restricting the scope of the model to a particular aspect of the problem, other methods are needed to model phenomena grasping their whole complexity. Health care is plenty of examples of this problem, such as the assessment of system efficiency considering together the performance of hospital, home-care, day-care, and social services; or the development of a case-mix which includes clinical severity, functioning, burden, support needs and available technical aids.

Modelling the complexity as a whole is really useful to provide reliable information to the decision-maker to improve both understanding of the target phenomena, and system planning or resource allocation through evidence-informed decision making. The assessment of complexity in the health sector has been recently forwarded [4,5]. However references are mainly based on the qualitative approach [4]; for that reason, advances on the capacity of modelling complexity from a wider approach incorporating also quantitative skills is of high interest in health care systems.

Within healthcare systems, mental health deserves special attention. First the development of early models of integrated care started in the mental health sector and in intellectual disabilities earlier than in most other areas in Medicine [6]. Complex community care systems comprising health, social and educational services as well as a balance across residential, outpatient and day care were planned in the 1950's in the UK [7] and in the early 1960's in the US [8]. As a matter of fact, the problems faced by the mental health sector are central to long term care for chronic medical conditions or for persons with disabilities [9]. Therefore mental health care may not be an exception, but the paradigm of complex integrated healthcare, as it was pointed out by Leon Eisenberg 35 years ago [10].

Mental health care, regarded as a holistic system, involves a very complex structure and poses a major challenge for modelling under the classical approaches, as occurs with most very complex phenomena.

As said, when a phenomenon is very complex, the classical modelling methods based on algebraic formalism cannot capture the whole structure of the domain and poor results are obtained [11]. Recently, some new approaches are defending the advantages of incorporating prior expert knowledge into the analysis itself [12,13]. Generalizing classical data analysis methods to be guided by prior expert knowledge permits a better modelling of these phenomena and improves the quality of the results.

Taking the prior expert knowledge into account is not necessarily related to a loss of scientific rigour, since, from the beginnings of the Artificial Intelligence in the mid 1950 s, there are strictly rigorous frameworks, based on the logical paradigm, to handle expert knowledge in a formal and automatic way [14].

This paper introduces a new methodological approach called *Expert-based Cooperative Analysis *(EbCA) as a general framework suitable for research in very complex medical problems, where classical approaches provide poor results. EbCA is based on formally including expert knowledge in the analysis. This proposal is, in fact, a methodology for generalizing any classical analysis method to incorporate expert knowledge as an essential part of the data analysis. This approach provides more powerful tools for better modelling complex phenomena, as those occurring in health systems. EbCA constitutes the main contribution of this paper and it is intended to contribute to a better understanding of integrated care as a complex adaptative system [2], overcoming the results obtained with classical approaches up to now.

The applicability of EbCA to two very different case studies is introduced as an illustration of both the suitability of the methodology in improving complex modelling and supporting complex decision making, and to illustrate the versatility of the proposal which could be applied in a wide range of data analysis methods and study designs.

## Methods

The authors have developed the *Expert-based Cooperative Analysis *(EbCA), a systematic procedure to incorporate expert knowledge into data analysis. This methodology has been applied in two very different cases using two well established analysis methods: "Clustering (ClBR) and "Data Envelopment Analysis" (DEA), which are described below. First, we present two case studies of complex mental health care carried out by the PSICOST research association that will later illustrate the benefits of the Expert-based Cooperative Analysis (EbCA) approach. Second a definition of EbCA is given. Third, we provide a detailed description of the main components and steps of this methodology: data preparation, expert knowledge transfer (including the formalization of this knowledge), and generalization of classical methods to involve prior expert knowledge. The EbCA approach is then applied to the data analysis methods used in the two case studies: *Data Envelopment Analysis *(DEA), and *Clustering *(ClBR) These two examples come from different environments: operational research and multivariate statistical analysis, and they illustrate the suitability of the EbCA methodology to improve the quality of results using widely different methods. New data analysis methods are obtained (*EbCA-DEA *and *Clustering based on rules or ClBR) *both including hybridation with Artificial Intelligence (AI). EbCA-DEA has been used to assess technical efficiency of health care areas (case study 1) and ClBR has been used to generate a case-mix of schizophrenia based on functional dependency (case study 2). Finally, we present conclusions and future work.

## Case studies

### Case study 1: Benchmark of small mental health care areas based on technical efficiency

Mental health care is a typical example of a very complex system where finding good global models is quite difficult under classical approaches. Mental health is organised in catchment areas comprising hospital care, residential care in the community, outpatient, emergency and different types of mobile care, as well as day care including occupational training, employment and other social services [6]. The assessment of integrated care systems at local areas may benefit from the identification of a benchmark area, and it should incorporate standard procedures for comparing efficiency across the different catchment areas. However, it is not always possible to ground decision making on actual efficiency based on population outcome indicators, particularly when information is incomplete, the units of analysis are complex and the outcomes refer to ill-defined conditions based on constructs such as mental disorders. *Technical efficiency *uses proxy measures to assess the proportion of outputs produced (e.g. hospital bed utilisation) related to the resources available (i.e. hospital bed availability).

We assessed the technical efficiency of 12 widely different small health areas (SHA) in Spain, in order to provide a new decision support system (DSS) for mental health planning in Spain. All existing health and social services for mental health care were mapped in every local area by an external rater using the European Service Mapping Schedule (ESMS) [15], as well as an Expert-driven Model of Basic Mental Health Community Care (B-MHCC) [13].

The data for each catchment area were aggregated in residential care, structured day care, non-acute out patient care and emergency out patient care, as it is shown in table 1. The detailed characteristics of the database have been described in [13].

**Table 1:** Operational description of integrated community care in Mental Health according to the European Service Mapping Schedule (ESMS) code groupings of "Main Types of Care" and technical characteristics of the Expert-driven Basic Model of Mental Health Community Care (B-MHCC) based on expert knowledge and characteristics of the 12 Small Health Areas (12 variables relevant for integrated community care have been included in the B-MHCC) (Salvador-Carulla et al, 2007) [9]

Grouping of services	ESMS Coding^(*)^	Description of ESMS "Main Types Care"	Variables: Types (T)^1^, Utilisation (U)^2^, Places (P)^3^	Technical characteristics of the Expert-driven Model of Community Care (B-MHCC)[rates per 100.000 population]^4^
Acute Care	R2	Residential/hospital/Acute	Types: TR2 Places (beds): PR2 Utilisation: UR2	High availability and utilisation by users from the area BUT avoiding over-use. 1. TR2 within a [1,1.5] range. 2. PR2 within a [9,20] range. 3. UR2 Medium-High to High avoiding over-use. Within a [10,19] range. T9 0[6], T1 (6,19], T9 (19,100]. U_w_[0.10,0.15]

Non-acute hospital care	R4, R5, R6 and R7	Residential/Hospital/Non-acute	Types: TR4R7 Places (beds): PR4R7 Utilisation: UR4R7	Low availability and utilisation BUT not "0". 4. TR4-R7 Low, within [1,3.1]. T9 [0,1.9], T1 (1.9,3.1], T11 (3.1,15]. U_w_[0.20,0.25]. 5. PR4-R7 Low, within [3,13]. T9 0[3], T1 (3,13], T7 (13,200]. U_w_[0.10,0.15]. 6. UR4-R7: Low use, within [3,12]. T9 0[3], T1 (3,13], T9 (13,100]. U_w_[0.13,0.17].
Residential community care	R8, R9, R10, R11, R12 and R13	Residential/Non-hospital	Types: TR8R13 Places (beds): PR8R13 Utilisation: UR8R13	High availability and utilisation. 7. TR8-R13: High [> 3]. T9 0[3], T2 (3,20]. U_w_[0.18,0.23]. 8. PR8-R13: High [> 10]. T9 0[3], T1 (3,13], T8 (13,20]. U_w_[0.10,0.15]. 9. UR8-R13 High avoiding over-use, within [10,40].
Day care	D1+D4^5^	D1: Day care/Acute (day hospitals) D4: Day care/Non-acute/Other structured activities	Types: TD1+D4 Places: PD1+D4 Utilisation: UD1+D4	High availability and utilisation 10. TD1+D4 High [> 3]. T9 0[3], T2 (3,20]. U_w_[0.25,0.3]. 11. PD1+D4 High [> 34]. T12 0[28], T11 (28,100]. U_w_[0.025.0.05]. 12. UD1+D4 High [> 33]. T9 0[15], T1 (15,37], T2 (37,100]. U_w_[0.13,0.17].

(*) For an explanation of ESMS service coding system see [26]. ^(1) ^T: Number of the ESMS codes or "main type of care" (R2, R4 to R7, R8 to R13 and D1+D4) per 100.000 inhabitants available within a small health area; ^(2) ^U: Utilisation per 100.000 inhabitants of the ESMS codes or "main type of care" by patients from the small health area; ^(3) ^P: Places or beds available per 100.000 inhabitants at the small health area; ^(4) ^Selected variables, T1, T2,... T12: I/O interpretation in a specific range, U_w_: Uniform statistical distribution of the I/O weight; ^(5) ^Work (D2), work-related (D3) and non-structured care (D5) have not been included in this

These data, together with prior expert knowledge, were used in EbCA-DEA to provide a ranking of the technical efficiency of the different systems. Separately, an expert panel made by 6 members provided a ranking of the small health areas based on full available data at the 12 areas. This allowed a comparison between both approaches to the analysis of the same set of data.

### Case study 2: Case-mix of schizophrenia based on functional dependency

In the 1990's the Council of Europe defined 'dependency' as the condition related to the loss of autonomy and the need of support from a third person due to impairment of activities of daily living, especially self-care. Laws and care services for the elderly and for people with severe disability including severe mental illness have been developed following this paradigm in many European countries, as well as eligibility criteria to different levels of care provision. Unfortunately current approaches have failed to provide a workable case-mix related to functional dependency, particularly in severe mental illness [16]. This is partly due to the complexity of the concept of *functional dependency *which has been described as a meta-construct involving the constructs functioning/disability, personal support and care needs [17], the different indicators related to these constructs (clinical status, functional impairment, quality of life, objective and subjective burden, service use, care needs, etc); and the additional complexity of mental disorders where disabilities are not just related to activities of daily living but to other aspects of general functioning such as social isolation, low medication adherence and behavioral problems that require intensive surveillance by carers [18].

In 2006 the Catalan Agency of Dependency commissioned a study aimed at improving the elegibility criteria of patients with severe mental illness to obtain specific benefits and care services within the context of the *Law for the promotion of personal autonomy and care for persons with dependency (LPAD 39/2006, 14^th ^december)*, approved by Spanish government in 2006 and enacting from 2007. This study was carried out by the PSICOST research association and the DEFDEP (DEFintion of DEPendency) group on a sample of 306 patients with schizophrenia [19] and it included the development of a case mix based on functional dependency. Patients were described by clinical subtypes and status, quality of life and functioning measured through the General Assessment of Functioning (GAF) [20] and the brief Disability Assessment Schedule (DAS) [21]. The care burden of family carers (performance in daily activities, behaviour, time and out-of-pocket expenditures) was measured using the Subjective and Objective Family Burden Interview (known by its Spanish acronym ECFOS) [22]. The utilisation of health and social care and care needs were also recorded. The database also contained clinical and demographic characteristics of the patients. This data, together with some prior expert knowledge was used for ClBR to model functional dependency patterns in schizophrenia.

## Data analysis method: Expert-based Cooperative Analysis

**Figure 1:**
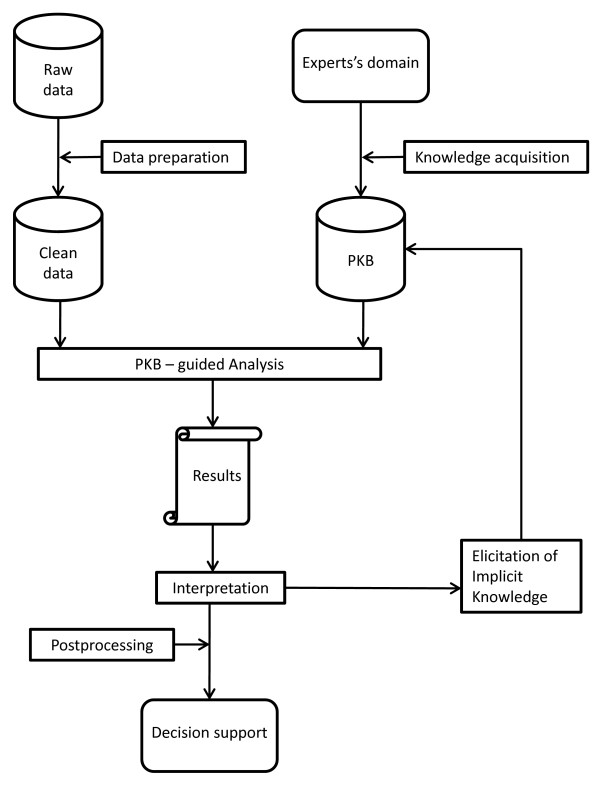
**EbCA-methodology**.

Expert-based Cooperative Analysis (EbCA) is a methodology which permits prior expert knowledge to be included in the data analysis and provides a general framework to iteratively elicit implicit knowledge from the experts. Previous independent experiences of the PSICOST research group [13,23,24] in Spain, based on different data analysis methods, resulted in successful modelling of highly complex phenomena related to health care system assessment. These separate studies showed a common analytical structure which differed from classical approaches and enabled a useful analysis of two complex domains for which classical techniques didn't succeed. This common structure has been formalized in the general EbCA methodology which includes both *Clustering based on rules (ClBR) *[12] and *EbCA-Expert-based Cooperative (EbCA-DEA) *[13] as particular cases. The EbCA is structured by the following steps (see Fig 1.) which are detailed in the next sections:

1. Data collection and Data preparation: includes selection of relevant variables to be considered and inclusion and exclusion criteria, data representation, missing data analysis, outlier detection and treatment, etc

2. Prior Expert Knowledge (PEK): acquisition and design of the Prior Knowledge Base (PKB).

3. PKB-guided analysis: PKB is used to guide the analysis in different ways depending on the underlying method selected

4. Support-interpretation: including tools to evaluate results and detect inconsistencies. In this step new Implicit Knowledge (IK) is elicited.

5. Incorporation of IK in PKB and repeat from step 3 until a satisfactory solution is found.

6. Post-processing results for decision support.

Direct involvement of the expert is crucial in steps 2 and 4.

### Data Preparation

The first step is not exclusive to EbCA, but it is necessary regardless of the analysis method used [25,26]. Data preparation is critical to decide:

• sample inclusion and exclusion criteria which determines the analysis units (set of patients, health areas etc),

• the set of variables to be considered for each analysis unit. Variable selection is an important challenge to deal with. It is critical to determine the set of variables that provide a relevant representation of the phenomenon under study. There are many different procedures for selecting relevant or *significant *variables, from statistical correlation [13] through multivariate analysis to artificial intelligence (AI) techniques like feature selection [27] or neural networks [28]. However, it is important to take into account the variable set the expert proposes to use, as this will often coincide with what he/she uses in real-life decision-making,

• the experimental/observational procedure or the reference data base.

These decisions should be followed by simple descriptive statistics, which [29] are useful to describe data and to get preliminary information from data.

Next, *data cleaning *and *data preparation *are fundamental steps to guarantee the quality of the original data, which is directly related to the quality and applicability of results. These processes include:

• missing data treatment

• outlier detection

• variable redundancy detection

• variable transformation (recodes, creation of indicators, etc)

The data preparation process must be conducted in close conjunction with the expert in order not to make false assumptions that can bias final results [30].

### Prior Knowledge Acquisition

To be formalized, explicit knowledge and implicit knowledge (IK) require different approaches. Explicit knowledge can be defined as the knowledge which is more or less directly available and can be explained by experts through standard tools like books, standard *If-Then *rules and so on [31]. On the other hand, IK, sometimes called *expertise *comes from people, places, ideas, experiences, habits or culture and cannot be found in books [32]. It remains hidden in the human mind and is unconsciously used in reasoning processes and unconsciously activated for decision-making, and is therefore extremely difficult to formalize. It is derived from the experience of the learner and is not directly available. It can be transferred to a system by using interactive approaches of knowledge engineering to help the expert to make his/her implicit knowledge explicit; machine learning techniques (from artificial intelligence)s can be used to automatically induce it from data; or the Knowledge Discovery from Data approach (KDD) can be used for extracting valuable knowledge from databases. As domain complexity increases, so too does the quantity of IK *implicitly *used in reasoning and decision-making and more powerful tools are required for elicitation.

Knowledge acquisition is the process of transferring expert knowledge from a human to a computing system. Knowledge Engineering appeared around the mid-1990 s as a part of the Artificial Intelligence discipline devoted to improve this process, as it has been seen the difficulties of the knowledge transfer process in complex phenomena. Knowledge engineering includes: i) transferring the correct contents to the computer; completeness and lack of ambiguity are critical here and the existence of IK must be taken into account, particularly in complex domains ii) expressing expert knowledge in such a way that the computer can understand it, a technical translation is required here and, finally, iii) the formal language used because each computer system has its own requirements and each methodological approach its own formalisms.

The knowledge that experts have in their minds -either implicit or explicit- is difficult to access and communicate, and is not easily shared [13,33]. However, expert knowledge is highly valuable and interesting to formalise, mainly for two reasons: i) once formalised, standard procedures can be established for decision-making and this facilitates the management of the domain (which requires knowledge transfer among professionals), and ii) once formalised, it can be transferred to a computer system and Intelligent Decision Support Systems can be used to provide on-line help in complex decision-making.

The expert-computer knowledge transfer process involves both explicit and IK and effective transfer is crucial for good computer system performance. When part of the expert knowledge is missing or transferred incorrectly, incomplete and inconsistent knowledge-bases (KB) result, which leads to faulty inductions in reasoning and wrong decision-making. Thus, while difficult, proper knowledge transfer is critical. If part of IK cannot be elicited, the computer system will ignore it and, therefore, the system will be unable to manage the reasoning involving it. Once elicited, the IK becomes explicit, and it is important to express this new explicit knowledge without ambiguities or inconsistencies.

The precise translation process of expert knowledge to computer systems becomes increasingly complicated because computer systems must understand the expert domain from an objective point of view, as they are unable to interpret informal definitions. Also, computers need deep expertise of the specific formal language to be used for modelling the domain knowledge [13]. The model in the computer system has to interpret expert knowledge in a dynamic way, using its own formal language. In this process knowledge engineers and experts in computer systems are translators.

Classical knowledge acquisition and transfer is a time consuming and costly process, mainly based on interviewing experts and refining the resulting models following an interactive knowledge engineering approach. Although they can miss part of the IK, these classical methodologies are useful to structure the skeleton of the knowledge-based system. Machine learning techniques provide alternative approaches, such as inducing rules from data, examples or empirical evidence, to model both explicit and IK using structured methods to analyse data [34]. These alternatives increase the efficiency of the knowledge acquisition process when data bases are available or can be designed and developed.

In the last few years, the advantages of using hybrid methodologies combining visualization, AI and statistical techniques for KDD and prior explicit knowledge have been highlighted [26]. This approach allows a well established explicit knowledge to be obtained from the experts, to build a partial but incomplete KB understandable to both the experts and computer systems, to combine a knowledge-based system with statistical techniques, to elicit IK, and to combine all into a final KB, which could be used in, for example, an operational model to guide technical analysis in specific decisional situations. Results obtained from these formal models can be used to improve the formal representation of expert knowledge in KB to design a new a more useful domain-model.

### Interpretation-support tools and PKB-guided analysis

Very often, when confronting complex phenomena, part of IK is missing in the first prior knowledge base (PKB) [7,11,13,23,25]. Since computer systems are not able to correctly reason with missing knowledge, poor results are produced. This is evidenced in the interpretation process. When non-sense results are obtained, the system works as an automatic knowledge acquisition tool. The knowledge engineer must then help the expert to find explanations for the apparently wrong results. This permits elicitation of IK and, in consequence new properties holding in target elements can be formulated, new relevant rules can be included in the PKB, new weights for the indicators/variables can be established or new combinations of inputs and outputs of the system can be considered. This permits reformulation of the PKB and improvement in results until they are clinically meaningful. Interpretation tools must support this process. Interpretation depends on the underlying analysis.

### Usability of EbCA in research

The EbCA approach has been incorporated in two very different methods (Data Envelopment Analysis and statistical clustering) in two separate studies of integrated mental health care: benchmarking and technical efficiency of integrated mental health care [13], and case-mix for schizophrenia based on functional dependency [24]. The units of analysis were small health areas in the first case and patients with schizophrenia in the second. Subsections for the main steps are also included. In both cases we compared the use of EbCA with classical qualitative approaches which formalised prior explicit knowledge provided by expert panels in a single run at the pre-processing phase. In case study 1 the agreement between the expert rating and the DEA model was estimated using a consistency model based on the Intra-class Correlation Coefficient (ICC) with a 95% Confidence Interval (CI) [35,36]. A Bayesian predictive validity analysis comparing expert judgment and DEA results was also performed. The 12 small health areas were grouped in two groups: *efficient *(efficient and nearly efficient small health areas) and *inefficient *(uncertain and inefficient small health areas). In case study 2 we used Cohen´s kappa to compare the case-mix developed by the expert panel and the ClBR, as well as Bayesian predictive measures.

## Results

We describe here the implementation of EbCA methodology in the two case studies mentioned above.

### Case 1: Use of EbCA in the analysis of healthcare systems by means of Expert-based Cooperative Analysis (EbCA-DEA)

We used Data Envelopment Analysis (DEA) for benchmarking small mental health areas using technical efficiency. DEA is a non-parametric method that evaluates the relative technical efficiency of a set of comparable Decision Making Units (DMU), each using multiple inputs to produce multiple outputs. DEA has been used in many decisional situations related to health care [13,37,38]. However, in these complex stochastic systems the application of DEA has some relevant drawbacks, such as: i) results obtained may not agree with the previous and well established experts' opinion [13], ii) inputs and outputs (variables in DEA models) are always stochastic and the selection of the appropriate statistical distributions (StDIS) to fit them is not a trivial matter and iii) the number of observations to be evaluated (relative technical efficiency) in the system is usually low compared to the number of "inputs and outputs" (I/O), which compromises the discriminative power of DEA models. In spite of these problems, prior expert knowledge has not been formalised in previous DEA studies.

EbCA-DEA incorporates explicit knowledge directly into the analysis by means of a Prior Knowledge Base (PKB) based, in this case, on standard *If-then *rules. Therefore, EbCA-DEA fits into the integral approach of Knowledge Discovery from Data (KDD), where prior expert knowledge (PEK) is taken into account at the beginning, and post-processing of results at the end, to lead to new explicit knowledge. Using this procedure more realistic (less simplified) scenarios can be analyzed in decisional situations under conditions of uncertainty and more useful models are obtained.

#### Data collection and Data preparation

According to our approach [13], the first step was to identify the decision making units (DMU) -in this case a series of comparable small health areas- to be analyzed, and to identify and evaluate the inputs and outputs (I/O) of service availability and utilisation that describe these DMU.

#### Expert-based selection of the stochastic structure of the variables

In uncertain environments, it is impossible to know the exact value of the variables because they are all random, sometimes with an unknown structure. EbC-DEA incorporates the uncertainty by using an expert-based statistical distribution for every I/O [39,40]. For example, *beds in acute units in general hospitals *(TR2 input, Table 1) can be fitted to a uniform distribution *U [2,3.5] *for a specific small health area -DMU. Hence the classical data matrix where cells contain categorical or real values is modified to include probabilistic models.

#### Expert-based variable selection: designing variable (I/O) combinations (scenarios)

Operational models, like classical DEA, can usually manage a limited number of variables [41,42]. However, when the number of variables is too large, the statistical elimination and/or reduction of some of them is arguable [43]. Nowadays, the analysis of all/some scenarios defined by different variable combinations -correlated or not- is more appropriate for EbC-DEA in complex systems [13]. These scenarios can be expert-based ones (i.e. experts select the I/O to be analyzed) or they can be automatically designed, (i.e. using a correlation analysis based on Monte-Carlo simulation). These two approaches show different technical views of the same problem and their combination can be used either to elicit experts' mental frameworks or to reach better conclusions about the efficiency of a specific DMU.

#### Prior Expert Knowledge (PEK) Acquisition, construction of Prior Knowledge Base (PKB)

EbCA-DEA needs a PKB to guide the analysis. According to our approach [13], a procedure derived from KDD is appropriate to assess technical efficiency. In this example, a consensus based *Expert-driven Basic Model of Mental Health Community Care *(B-MHCC) was designed and developed (Table 1) by an operational analyst and two experts. A technical report with a full description of the care system in twelve small health areas was used for *knowledge engineering *which consisted of developing and understanding the domain and capturing relevant prior and implicit knowledge from the experts. The resulting B-MHCC includes the expert-based structure of all the selected inputs and outputs (I/O) in each small health area (the decision making unit or DMU in this case).

In DEA models, a *standard input *is a variable such that the greater the value the lower the efficiency (holding the rest of the I/O values constant). On the other hand, a *standard output *is such that the greater the output the greater the efficiency. However, some I/O in complex systems may have an opposite meaning, as their high values are related to system inefficiency. Examples include *non-standard inputs*, such as a low availability of residential beds in the community; and *non-standard outputs *such as a high utilisation of indefinite-stay psychiatric hospital beds in the small health area. The B-MHCC contains the expert-based interpretation for all the I/O to determine if they can be considered standard or not (Table 1). In this framework, the PKB is based on standard *If-then *rules that translate expert knowledge to the computer system. When a non-standard I/O has to be evaluated, the PKB also includes information about the appropriate mathematical transformation that uses, in this case, a linear monotone decreasing transformation [44,45].

#### PKB-guided analysis

The selection of the appropriate operational model in EbCA-DEA is a result of a consensus between the operational modellers and the experts. Both researchers and modellers have to understand the consequences of applying a specific procedure and must share the same language [13,23] to be able to select from different Monte-Carlo DEA models, such as input oriented, output oriented, those that take into consideration constant returns to scale, and those that understand variable returns to scale, among others.

Once the DEA model is selected in the EbCA-DEA, Monte-Carlo simulation is performed to determine I/O values based on the probabilistic models provided by the experts, and a simulated data matrix is obtained. Then PKB is used to determine which I/O are standard and which are not, and to apply the corresponding mathematical transformations. Finally, DEA model is designed and resolved as standard linear programme. Each simulation evaluates the relative technical efficiencies of each DMU which is then saved in a pool of results. The process stops when the results converge and no significant improvements are achieved on DMU technical efficiencies regarding previous simulation runs. Therefore, the same DMU has different relative technical efficiencies depending on the I/O values selected by the Monte-Carlo engine.

#### Support-interpretation tools to evaluate results and detect inconsistencies: elicitation of implicit knowledge

Obviously, the greater the simulation runs performed by the Monte-Carlo engine, the larger the database containing the results. Then results are statistically analyzed. Different techniques can be used such as: agreement ratios [13], clustering procedures, and data mining. Potential agreements or disagreements between DEA results and the experts' background knowledge are used again for improving prior expert knowledge in the PKB. In this iterative process the understanding of the experts' mental framework is improved, and IK elicited to further refinement of the Basic Model of Mental Health Community Care (B-MHCC).

Even though DEA is useful to identify efficient areas, both inefficiency levels and the specific I/O related to inefficiency in every DMU are potentially debatable from expert's point of view [13]. This behaviour is related to the different interpretations of relative technical efficiency by experts, on one hand, and DEA models, on the other. DEA only takes into consideration the I/O values and compares different but comparable DMU. As it cannot evaluate the appropriateness of specific I/O profiles, a DMU can be technically efficient (DEA) but its I/O may be judged inappropriate by the expert.

The expert-driven DEA model produced 7 different scenarios. IK elicited from the I/O combinations helped to define these scenarios. Using this IK, now explicit, the B-MHCC was improved (incorporation of IK in PKB) and new and better efficiency-based classifications were obtained (Post-processing results for decision support). At the end of this iterative process a bench-mark area was identified and two input and four output indicators were selected out from a set of 74 variables (the PSICOST-74 indicator set) to assess health system efficiency in a basic model of mental health community care. Scenario ''6'' was identified as the best option [ICC: 0.8705; CI (95%): 0.5500; 0.9627]. The full description of these results is provided in [13].

However, when the ranking of small health areas using the EbCA-DEA model was compared with the rating obtained by the expert panel, an unexpected disagreement was found. Scenario "6" classified six areas in the ''efficiency'' group, but 3 of these areas were *false-positives *according to the experts' rating which was used as gold-standard. Six areas were classified in the ''inefficiency'' group by the EbCA-DEA while the expert panel identified 7 areas in this group. Therefore the DEA model agreed in five areas out of 12 with the expert panel using the best scenario. Compared to the expert panel rating, DEA showed a high sensibility (100%) and a low specificity (66.6%). The Positive Predictive Value (PPV) was 50% (IC: 13.9-86.0%) while the Negative Predictive Value (NPV) was 100% (IC: 51.6-98.4%). The Positive Likelihood Ratio was 3.0 (IC: 1.1-7.5) and the Negative Likelihood Ratio was 0.

Once the study was completed, the core group made a careful review of the results area by area which revealed that, in spite of the previous information provided to experts, their ratings were not based on efficiency but on adequacy of the small health areas. That is, experts judged as *efficient *or *nearly efficient*, those areas with high level of provision and resources (high adequacy) regardless of the fact that these areas were using their resources less efficiently than other areas with lower provision. This conceptual disagreement between the panel and the DEA model explained the poor PPV found in our study. Therefore the *gold standard *based on explicit prior knowledge provided by the expert panel and tested with regional officers revealed itself inadequate to rank technical efficiency of small health areas in comparison with the EbCA-DEA. When confronted to this fact, experts agreed that they had measured adequacy instead of technical efficiency. This fact may indicate that adequacy and technical efficiency should be measured together to avoid this conceptual bias, and it illustrates the usability of EbCA in Data Envelopment Analysis for the assessment of benchmark and technical efficiency of complex healthcare systems.

### Case study 2: Use of EbCA in case-mix of schizophrenia based on functional dependency by means of Clustering based on rules (ClBR)

In case study 2, we used Clustering Based on Rules (ClBR) to develop a case-mix of schizophrenia based on the meta-construct of "functional dependency". ClBR is a technique in [12] which provides operational definitions for underlying profiles in a very complex domain. It guarantees the semantic meaning of the resulting classes, which is extremely useful for later decision support. ClBR is a hybrid AI and Statistics technique that combines some Inductive Learning (from AI) with clustering (from Statistics) to extract knowledge from complex domains in the form of typical profiles. A Knowledge Base (KB) expressing the existent prior domain knowledge is considered to properly guide the database clustering. It is important to note that the KB allows declarative knowledge to be used in a formal way. ClBR is implemented in the software KLASS [26]. An important property of the method is that quantitative, qualitative and declarative knowledge is processed and the semantic constraints implied by the KB are held in final clusters, which guarantees the interpretability of the resulting classes. It improves classical clustering because results are semantically consistent and, consequently, easier to interpret [12]. It outperforms pure inductive learning methods, since it reduces the effects of missing some IK in the KB.

ClBR fits into the integral approach of KDD, [46] in which prior expert knowledge (PEK) and post-processing of the analysis results are taken into account, producing explicit knowledge directly understandable by the expert [11,47]. As a matter of fact in this approach the expert is regarded, as part of the methodology itself.

#### Acquiring prior expert knowledge

After a proper and detailed data preparation, acquisition of PEK was performed by means of classical knowledge engineering techniques, following a series of interviews between experts and knowledge engineers with two basic goals:

• Identify what is well-known in the domain: for example, properties of some kinds of patients and relationships between different variables under some circumstances, among others. At this stage, classical knowledge engineering requires a *complete *description of the domain, without missing any details or exceptional cases. This is one of the most difficult tasks, since complex phenomena imply an enormous amount of IK that requires many iterations to become explicit. In this approach, a partial description is enough, provided it does not include ambiguities and inherent contradictions. Incremental elicitation of IK is allowed under a series of iterations when required. Previous experiences shown very good results when starting the process by asking the experts for an a priori description of what is called the *basic extreme antagonism*, i.e. the description of a prototypical worst-case and a prototypical best-case. For example, as the functional dependency profiles have to be discovered using a well constructed set of patients, the starting questions for experts were:

◦ *What characterises a person with schizophrenia and the highest level of dependency?*

◦ *What characterises a person with schizophrenia and the highest level of functional autonomy?*

Although this is a good practice for using ClBR, basic extreme antagonism is not the only way to build the prior expert knowledge base in first iteration. If there are well-known prototypical cases regarding the target domain, they may be included in the first PKB as well, and this can avoid some iterations of the process. However, it is worth to note that the efficiency of the method is related to the discriminant power of the PKB rules and the quality of the results is not monotonically related with the size of PKB. This means that using a high number of non-well discriminable prototypes may decrease the goodness of the results, whereas limiting first PKB to the basic extreme antagonism guarantees maximum discriminant power of the rules and avoids some risks.

• From the first iteration, experts are able to provide several rules concerning some clear situations. Rules corresponding to the extreme antagonism are: patients with bad levels of functioning (GAF), high needs of family support in daily activities (ECFOS section A) and behavioural problems (ECFOS section B) can be considered as severely ill

• patients able to work and with high levels of functioning (GAF) can be considered as patients in good condition.

• To formalize the identified knowledge in an initial knowledge base (a set of rules expressed in formal logics under the form *If <condition> then <property> (or <group identifier) >)*

◦ r1: *If *((GAFCLA < 40) or (GAFSOCIAL < 40))*and*((MAXECFOS_A > 15) *and*(MAXECFOS_B = Every_Day)) *then *severely-ill

◦ r2: *If *(INGRESE = WORK) *and *(GAFCLA > 70)or(GAFSOCIAL > 70) *then *good condition

Meetings between experts and knowledge engineers within the methodological frame of knowledge engineering produced the first knowledge transfer to the system by making an initial set of formal logic definitions which, of course, constituted an incomplete description of the phenomenon. In the proposed approach, instead of working hard to arrive at a complete description initially, an iterative methodology permits incremental improvements of PKB until only non-relevant gaps are detected.

#### Support-interpretation tools

**Figure 2:**
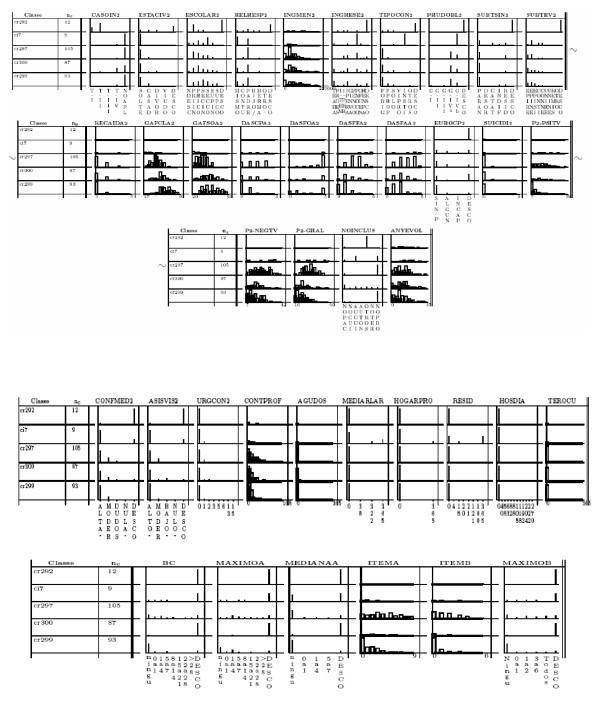
**Class panel graph**. Every row shows one of the classes resulting from the ClBR process. In the columns, variables (relevant characteristics of the patients or items from the assessment scales) are presented. Every cell of the table shows the distribution of a given item or characteristic in a certain class This permits to identify wich variable have particular behaviour in which classes. Details of the interpretation iduced from these class panel graphs are presented in [12].

The results are graphically represented in a dendrogram (a binary tree which visualises how patients have been successively grouped by the method and permits the identification of the suitable number of profiles). The Calinski index [48] is maximized upon the dendrogram to determine the number of classes on the basis of what is suggested by the structure of analyzed data itself, rather than a priori supposing a blind number of classes as occurs in other clustering methods. Interpretation of the classes was formerly difficult and time consuming and required much human guidance, particularly in cases with many relevant variables or many final classes. Efforts to assist experts in this task led to the battery of interpretation-support tools used here [47,49]. First, for all the variables, the relevance of differences between classes is assessed using the corresponding statistical test (ANOVA, Kruskall-Wallis test or chi-2 independence test). Then a class panel graph (CPG, Fig. 2) of significant variables is charted, where conditional distributions of the variables through the classes, displayed through multiple histograms or barcharts, are shown in a compact way [49]. The knowledge engineer marks the more characteristic cells of the CPG. Experts use the marked CPG to easily observe why the variables are significant and relevant for the description of the profiles; particularities of the variables in one specific class regarding the others can be seen and evaluated by the experts and, as non incoherencies are found, this helps the expert to develop a *conceptualization *process which leads to a class-labelling proposal regarding the semantic entity represented by each class.

#### Identifying profiles by ClBR

The *Prior Expert Knowledge *PEK acquisition phase produces a first short and *incomplete Prior Knowledge Base *PKB (of course we can find other patients in good conditions who do not satisfy the second rule, they may, for example, not work, just because their family is wealthy). PKB is used to guide a hierarchical clustering process (in particular a reciprocal neighbours algorithm with Ward's criteria and Gibert's mixed metrics [50]): it is evaluated over the sample, and patients satisfying every rule are identified and pre-processed together; a prototypical description of patients satisfying every rule is automatically induced and used to be clustered with the remaining patients. This produces a global partition of target patients, coherent with PEK, including those that satisfied PEK and the rest.

Support-interpretation tools were used at every iteration to help experts conceptualize the identified groups. Some iterations are often required to gradually complete the PKB with relevant elicited IK and to obtain a set of profiles which can describe the whole set of patients through homogeneous groups of patients that are distinguishable from each other and that provide a set of distinctive characteristics for each group, thus giving a semantic explanation of the results. This explanation contributes to an operational definition of functioning in schizophrenia and can be used to better understand dependency patterns in our immediate environment and to support proper decisions about resource allocation and planning. For example, in the specific case of Spain, decisions are related to the implementation of the Spanish Law of Dependency and the use of the official assessment tool for eligibility and classification of persons with dependency.

#### Resulting profiles

The first PKB contained the two rules presented above. Some iterations were required to elicit the expert IK required for completing the analysis. As part of the process several contradictions were evidenced, and these where used to improve the PKB for next iteration. As an example, in first iteration a class was conformed with highly dependent persons. However, the family care needs of the class showed intermediate values with was not intuitive at all. Going further on the analysis of the class composition it could be understood that it contained both patients living in the community and some staying in institutions. The former had very high family care needs, while the later benefit from institution's resources and registered very low family care loads, thus producing a non-sensical intermediate values for family care needs in this class. A new rule could be introduced to identify institutionalized patients and then, the meaning of the resulted classes improved. Five iterations with different improvements were required to reach final results. Finally, 5 classes with different patterns of functioning and increasing levels of functional dependency were identified, these were all clinically understandable from the medical point of view. The proposed interpretation-process provided a conceptual interpretation of the classes [24].

The analysis identified a group of patients with missing information (drop-outs).

A second group of *Autonomous persons*, without functional dependency in spite of their illness, could perform activities of daily living (ADLs) without surveillance, were generally employed; and had a shorter course of illness than other groups.

Patients with functional impairment were subdivided into three different profiles according to their dependency level: those who lived alone, those highly impaired living with their families, and those in long-term residential care [24]. Particularly interesting for the experts was the elicitation of a *Living Alone *group. These patients did not have high dependency levels and had no severe impairment for performing strict ADLs, but did show significant behavioural problems; tended to have inadequate monitoring/surveillance and poor treatment adherence, and also showed inappropriate use of services (*i.e*. missing scheduled visits and using emergency service as their main care resource, some of them up to 50 times per week). Notably, the parallel expert panel did not identify the *Living Alone *profile discovered by ClBR, although all experts agreed upon the relevance of this class when presented as part of the ClBR results. The kappa agreement between both classifications was low (kappa: 0.12) although it improved after incorporating the "Living alone" group to the classification of the final consensus panel (kappa: 0.58). Compared to the final panel classification, the revised ClBR showed a sensitivity of 67.7%, a specificity of 82%, a Positive Predictive Value (PPV) of 71.9% (IC: 62.6-79.7%), a Negative Predictive Value (NPV) of 78.9% (IC: 72.1 - 84.4%), a Positive Likelihood Ratio of 3.7 (IC: 2.6-5.2) and a Negative Likelihood Ratio of 0.3 (IC: 0.3-0.5).

## Discussion

This paper proposes a new methodological approach, named Expert-based Cooperative Analysis (EbCA), which provides a new paradigm where expert knowledge can be formally included in any data analysis method to obtain high quality results.

Modelling very complex phenomena, as healthcare systems, is a very difficult task, especially if resulting models have to provide proper support to real decision-making.

Until recently, there were no formal tools to analyze the knowledge in its declarative form, and the restrictive experimental approach was the most rigorous and powerful method for scientific research. However, the classical experimental approach, strictly based on collected data and techniques relying on algebraic fundamentals may not capture the whole complexity of the intricate phenomena found in real practice. Some trials to include prior expert knowledge provided some advances in this topic:

Operational models, which are based on more or less specific mathematical and/or statistical procedures, as well as other analysis techniques which use prior knowledge try to solve decisional situations that can be modelled using formal procedures and well-known algorithms. Stevens and O'Hagan [51] state that, "submissions of evidence... using the Bayesian approach must include supporting documentation that demonstrates clearly that a formal process of elicitation has been followed if the prior information is to be accepted as credible." The importance of incorporating both explicit and tacit knowledge has also being stated when using structural equation modelling [52]. However, none of those techniques can treat the prior knowledge in its declarative form. Some of them transform into distribution probabilities (bayessian approaches) or into algebraic equations, what represents a serious limitation on the kind of knowledge that can be expressed and incorporated into the analysis. On the other hand, owing to this translation required between the knowledge expressed by the experts and their introduction into the analysis, those techniques usually gather prior knowledge just at the pre-processing phase and a separation persists between the expert knowledge transmission to the system and formalisation and data analysis itself.

EbCA, as initially structured by our group [13], constitutes a new possibility of including prior knowledge into the analysis, more powerful and flexible than other previous alternatives because it takes advantage of the new formal frameworks provided by Artificial Intelligence to deal with declarative knowledge under symbolic paradigms, and permits to take advantage of the high value of existing expert knowledge, under his natural form. EbCA is suitable to evaluate the complexity of health care systems. Incorporating this knowledge into data analysis contributes to improve complex decision-making, rather than decrease the scientific rigour in research, provided this knowledge is included in the analysis using proper formalism.

On the other hand, the use of formalisms which do not require hard formalizations of expert knowledge (such as to transform them into probability distributions or algebraic equations) permits to eliminate one of the strongest limitations of classical knowledge-based methods and other prior knowledge based methods (such as bayessian methods or structural equations). All those approached seriously depended on the *completeness *of the prior knowledge transmitted to the system, at the beginning of the data analysis process. It is obvious that as implicit knowledge was not included in those prior knowledge descriptions the performance of the methods were seriously damaged.

EbCA is dealing with prior knowledge without hard transformations, it keeps in declarative form and do not require hard pre-processing of the prior knowledge to include in the data analysis. This makes possible to use an iterative scheme where, the completeness assumption for the prior knowledge can be eliminated. Partial prior knowledge can be considered at the beginning of the process and a sequential dialogue between experts and knowledge engineers is established that guides the cooperative incremental generation of new knowledge as well as elicitation of implicit knowledge along the whole process. Under this approach, expert knowledge can be included formally and incrementally in the analysis improving quality of results. The underlying mechanism is simple, but highly effective: cooperation between automatic PKB evaluation (relationships between variables or individuals in ClBR or rule evaluation in EbC-DEA) and blind analysis (hierarchical clustering- in ClBR or DEA in EbC-DEA) permits the completion of an initialy partial description of the domain provided by the experts to get good models for complex phenomena.

Whatever the data analysis technique being used, EbCA may provide the frame for generalizing it in such a way that experts can introduce their explicit knowledge into the analysis and, using the suitable interpretation support tools, elicit implicit knowledge which was previously disregarded, learning from the results of the analysis, and permit its immediate incorporation into the model. Finally, the relevant knowledge for the decisional problem to be solved is elicited, extracted and formalized. Being supported by an analytical method, this new knowledge can be used to improve decision making process based on informed-evidence.

EbCA methodology fits into the general framework of KDD where both the prior knowledge and the post-processing of results are as important as the analysis itself to produce useful and understandable models [46,53]. A significant contribution of EbCA is the use of standard strategies to improve knowledge transfer from experts and decision makers to the knowledge engineers and vice versa, using a common language. As a matter of fact, the use of EbCA approach may be regarded as the first step in the development of ontologies for very complex domains which may help to structure the doctrinae corpus and therefore improve decision making [54]. In knowledge engineering it is usual to develop ontologies from scratch, using expert interviews as the source of expert knowledge. However, the risk of getting incomplete ontologies due to implicit knowledge increases significantly with the complexity of the target phenomenon. The EbCA approach may improve the development of comprehensive ontologies in fields where knowledge is disperse and/or incomplete. EbCA-DEA and ClBR show the feasibility of using EbCA to incorporate expert knowledge into other data analysis techniques. Both ClBR and EbCA-DEA may be regarded as hybrid or mixed qualitative-quantitative techniques (knowledge management, knowledge engineering and data analysis) which combine classical data analysis techniques and AI methods that permit prior knowledge processing [30]. These two techniques have proven to be more powerful than the corresponding classical methods (hierarchical clustering or classical DEA) for modelling complex domains.

EbCA has been applied to other areas of medicine relevant to integrated care. Particular methods following the EbCA approach have been used to identify case-mix in traumatic brain injury based on the characteristics of deficit and response to neuro-rehabilitation treatment [11], to describe patterns of evolution over time of the Quality of Life (QoL) of patients with spinal cord injury attended in integrated [55], to the analysis of integrated home care support system [56], to find profiles of patients with thyroid dysfunctions [12], to find profiles of functional disability in elderly patients on the basis of the information provided by the WHO-DAS [57], or to identify vulnerability factors to develop comorbid mental disorder in an intellectually disabled population [58]. Some of these studies had real impact in the integrated care and decision making. As an example, the use of ClBR in brain injury [11] identified which kind of patients shown partial response to the neuro-rehabilitation treatment and which were the non-rehabilitated neuropsychological functions so that the hospital managers could take decisions regarding the neuro-rehabilitation programs for those patients [55]. It also elicited hypothesis about the reasons why patients develop distress or depression, thus helping on better coordinated intervention planning between doctors, psychologists, social workers and relatives aimed to avoid (or delay) them and [57] evidenced that functional disability in ageing patients is not always due to physical impairment, but to emotional problems as well, so treatment programmes could be designed accordingly.

## Conclusions and Future Work

Taking advantage of the well-known prior expert knowledge and adopting an iterative strategy to help the expert to elicit the extensive implicit knowledge that he or she uses in daily reasoning and decision-making is a powerful method which should be considered a new research paradigm to improve medical *doctrinae corpus*. This approach provides an operational method to bridge and transfer knowledge between clinicians and data analysts when studying complex entities such as integrated care. Examples of its applicability in integrated care for persons with severe disabilities [11,13,24,55,56,58] and ageing [56,57].

In the future, usability of EbCA should be tested to generalize other analysis methods, as the addition of implicit knowledge to classical techniques may improve the quality of results regardless of the underlying method. Impact assessment studies are required to explore the usability of this approach in comparison to classical techniques of data analysis. Further application of EbCa approach to health service research might produce significant improvements in better understanding integrated care systems and in developing evidence-based policy and planning in this area.

## Competing interests

The authors declare that they have no competing interests.

## Authors' contributions

The three authors worked together in the development of the model. CG and LSC applied it to DEA (case 1), and KG and LSC applied it to ClBR (case 2). The three authors wrote the paper. All authors have read and approved the final manuscript.
